# Supplementary material for: Do xenophobic attitudes influence migrant workers’ regional location choice?
Source: PLoS One. 2025 Feb 5;20(2):e0316627. doi: 10.1371/journal.pone.0316627 (PMC11798449; doi:10.1371/journal.pone.0316627)
Supplement: S1 Table — (DOCX) [file pone.0316627.s001.docx]

**S1 Table A1: Variable definitions and data sources**

| **Dependent variable** | **Definition** | **Source** | **Period** |
| --- | --- | --- | --- |
| Immigration rate | Sum of immigrants and regional workforce divided by regional workforce | Integrated Employment Biographies (IEB) | 2001-2018 |
| **Pivotal explanatory variables** | | | |
| Politically motivated right-wing crime | Number of politically motivated offenses by right-wing extremists per 1,000 employees | Federal State Criminal Police Offices | 2001-2019 ^(a)^ |
| Right-wing votes | Share of votes for right-wing parties in federal elections | Federal returning officer (Bundeswahlleiter) | 2005, 2009, 2013 ^(b)^ |
| **Control variables** | | | |
| **Economic and labour market conditions** | | | |
| Wage level | Gross annual wage per capita (in 1,000 EUROs) | National accounts of the Federal States | 2000-2017 |
| Unemployment rate | Number of unemployed persons divided by labour force (in %) | INKAR database of the Federal Institute for Research on Building, Urban Affairs and Spatial Development (BBSR) | 1999-2017 |
| Employment growth | Annual growth rate of employment | Employment statistics of the Federal Employment Agency | 2002-2017 |
| Sector shares | Share of sectors in total employment for top 10 industries in which immigrants are employed | IEB | 2000-2017 |
| Public financial capacity | Fiscal capacity of municipalities per capita in thousand EUROS | INKAR database of the Federal Institute for Research on Building, Urban Affairs and Spatial Development (BBSR) | 1999-2017 |
| Social welfare rate | Share of welfare recipients in total population < 65 years | INKAR database of the Federal Institute for Research on Building, Urban Affairs and Spatial Development (BBSR) | 2000-2016 |
| **Housing market** | | | |
| Flat size | Average flat size per inhabitant in m² | INKAR database of the Federal Institute for Research on Building, Urban Affairs and Spatial Development (BBSR) | 1999-2017 |
| Land price | Average land price (land ready for building) in EUROS per m² | Regionaldatenbank Deutschland of the Federal Statistical Office of Germany | 1999-2016 |
| **Amenities and Disamenities** | | | |
| (Spatial lag of) Population density | Inhabitants per km^2^ (in 1,000) | Regionaldatenbank Deutschland of the Federal Statistical Office of Germany | 2000-2016 |
| Share of foreign population | Share of foreign inhabitants in total population | INKAR database of the Federal Institute for Research on Building, Urban Affairs and Spatial Development (BBSR) | 1995-2017 |
| Share of creative economy | Share of cultural industry and creative economy in total employment | IEB | 1999-2017 |
| Overnight stays | Number of overnight stays per inhabitant | Regionaldatenbank Deutschland of the Federal Statistical Office of Germany | 1999-2017 |
| Recreation area | Recreation area divided by the total area in % | Regionaldatenbank Deutschland of the Federal Statistical Office of Germany | 2000, 2004, 2008-2016 |
| Crime rate | Number of cases (street crime) per 100,000 inhabitants | Crime statistics of Germany`s Federal Criminal Police Office (BKA) | 2003-2016 |
| **Further control variables** | | | |
| Voter turnout | Share of voters among eligible voters, Bundestag elections | INKAR database of the Federal Institute for Research on Building, Urban Affairs and Spatial Development (BBSR) | 2002, 2005, 2009, 2013, 2017 |
| **Instrument variables** | | | |
| Vocational training | Ratio of vocational training positions to graduates demanding training | INKAR database of the Federal Institute for Research on Building, Urban Affairs and Spatial Development (BBSR) | 1999-2017 |
| Longitude | Longitude of the largest city in the count region | Regionaldatenbank Deutschland of the Federal Statistical Office of Germany |  |
| Low-skilled foreign workers | Share of low-skilled labour among foreign workers | Employment statistic of the Federal Employment Agency | 1995-2017 |
| Foreign population | Share of foreign population in total population | INKAR database of the Federal Institute for Research on Building, Urban Affairs and Spatial Development (BBSR) | 1995-2017 |

1. Information on politically motivated right-wing crime is not available for the entire period in all district regions, because federal states started to report the figures at different points in time. Schleswig-Holstein: from 2015 onwards, Hamburg: 2010, Lower Saxony: 2011, Bremen: 2005, North Rhine-Westphalia: 2006, Hesse: 2001, Rhineland-Palatinate: 2010, Baden-Wuerttemberg: 2014, Bavaria: 2010, Saarland: 2009, Berlin: 2003, Brandenburg: 2001, Mecklenburg-Western Pomerania: 2004, Saxony: 2010, Saxony-Anhalt: 2007, Thuringia: 2009. Due to these data restrictions and a few missing observations for land prices and the share of temporary employment agency the number of observations used in the corresponding regression analysis amounts to 2,778 (fixed effects model) and 2,772 (fixed effects instrument variable estimation) instead of 5,040 observations (14 x 360) in case of a balanced sample, see also Table 2.
2. Information on the share of votes for right-wing parties in federal elections is available for 2002, 2005, 2009, 2013 and 2017. As immigration data is available for the period 2004 to 2017 and all regressors enter with a time lag, we can only include the election results in 2005, 2009 and 2013 in the regression analysis. The maximum number of observations for this specification is thus 1,080 (3 x 360 district regions). In the regression analysis with the election results being applied to measure xenophobic behaviour, the number of observations is 1.050 (see Table 1) due to missing observations for land prices and the share of temporary employment agency.
