# Supplementary material for: Do xenophobic attitudes influence migrant workers’ regional location choice?
Source: PLoS One. 2025 Feb 5;20(2):e0316627. doi: 10.1371/journal.pone.0316627 (PMC11798449; doi:10.1371/journal.pone.0316627)
Supplement: S2 Table — (DOCX) [file pone.0316627.s002.docx]

**S2 Table A2: Summary statistics**

|  | mean | sd | min | max |
| --- | --- | --- | --- | --- |
| Immigration rate | 4.310 | 3.344 | 0.270 | 38.313 |
| Immigration rate, skilled | 2.727 | 2.083 | 0.102 | 17.551 |
| Immigration rate, rest | 7.861 | 6.528 | 0.182 | 77.736 |
| Immigration rate, EU | 2.638 | 2.468 | 0.000 | 31.515 |
| Immigration rate, Non-EU | 1.474 | 1.186 | 0.039 | 9.651 |
| Rate xenophobic violence | 0.790 | 0.655 | 0.000 | 5.377 |
| Share right-wing votes | 0.088 | 0.062 | 0.005 | 0.376 |
| Wage level | 28.881 | 4.638 | 18.700 | 52.405 |
| Unemployment rate | 0.072 | 0.038 | 0.012 | 0.254 |
| Employment growth | 0.025 | 0.147 | -0.686 | 2.412 |
| Share of construction of buildings | 0.012 | 0.007 | 0.001 | 0.080 |
| Share of specialized construction activities | 0.047 | 0.015 | 0.011 | 0.124 |
| Share of land transport | 0.019 | 0.007 | 0.004 | 0.052 |
| Share of warehousing, support activities for transportation | 0.023 | 0.018 | 0.002 | 0.143 |
| Share of accommodation | 0.010 | 0.012 | 0.000 | 0.101 |
| Share of food and beverage service activities | 0.021 | 0.007 | 0.008 | 0.076 |
| Share of other professional, scientific and technical activities | 0.001 | 0.001 | 0.000 | 0.015 |
| Share of temporary employment agency | 0.025 | 0.016 | 0.000 | 0.155 |
| Share of services to buildings and landscape activities | 0.021 | 0.009 | 0.004 | 0.072 |
| Share of human health activities | 0.075 | 0.023 | 0.026 | 0.207 |
| Population density | 0.537 | 0.741 | 0.036 | 4.676 |
| Share of foreign population | 0.078 | 0.050 | 0.007 | 0.350 |
| Land price | 140.907 | 157.763 | 6.102 | 2099.154 |
| Recreation area | 2.259 | 2.742 | 0.199 | 14.956 |
| Share of creative economy | 0.029 | 0.016 | 0.006 | 0.134 |
| Overnight stays | 5.114 | 5.863 | 0.000 | 49.500 |
| Social welfare rate | 0.044 | 0.017 | 0.004 | 0.113 |
| Public financial capacity | 698.386 | 254.808 | 195.100 | 2611.400 |
| Voter turnout | 0.709 | 0.049 | 0.554 | 0.841 |
| Flat size | 46.449 | 4.956 | 35.100 | 63.500 |
| Crime rate | 1437.069 | 790.546 | 277.182 | 5813.474 |
| IV foreign population | -29.278 | 12.995 | -60.592 | -11.302 |
| IV supply vocational training | 0.003 | 0.051 | -0.181 | 0.253 |
| IV low-skilled foreign workers | -15.232 | 9.232 | -51.236 | -3.284 |

Notes: IV low-skilled foreign workers: lagged (7 years) log share of low-skilled among foreign workers interacted with longitude. IV foreign population: lagged (9 years) log share of foreign population interacted with longitude. IV supply vocational training: lagged (1 year) ratio of vocational training positions to graduates demanding training.
