# Supplementary material for: Do xenophobic attitudes influence migrant workers’ regional location choice?
Source: PLoS One. 2025 Feb 5;20(2):e0316627. doi: 10.1371/journal.pone.0316627 (PMC11798449; doi:10.1371/journal.pone.0316627)
Supplement: S3 Table — (DOCX) [file pone.0316627.s003.docx]

**S3 Table A3, Interpretation of control variables**

**Table A3: Instrument variable results for right-wing votes - controls and first stage**

|  | All | Skilled | Unskilled/unknown qualification | EU | Non-EU | First stage |
| --- | --- | --- | --- | --- | --- | --- |
| Share right-wing votes | -0.00764^***^ | -0.00569^***^ | -0.01283^***^ | -0.00569^***^ | -0.00084^**^ |  |
|  | (0.00204) | (0.00147) | (0.00386) | (0.00170) | (0.00032) |  |
| IV supply vocational training |  |  |  |  |  | -0.00642^***^ |
|  |  |  |  |  |  | (0.00193) |
| IV low-skilled foreign workers |  |  |  |  |  | 0.05683^***^ |
|  |  |  |  |  |  | (0.01408) |
| L.unemployment rate | 0.00822^***^ | 0.00490^***^ | 0.01250^***^ | 0.00675^***^ | 0.00085^***^ | 0.76603^***^ |
|  | (0.00206) | (0.00140) | (0.00407) | (0.00177) | (0.00030) | (0.07957) |
| L.wage level | -0.00222 | -0.00165 | -0.00530 | -0.00171 | -0.00016 | -0.31453 |
|  | (0.00256) | (0.00217) | (0.00544) | (0.00222) | (0.00050) | (0.25285) |
| L.employment growth | -0.00039 | 0.00018 | 0.00035 | 0.00029 | 0.00001 | 0.04519 |
|  | (0.00042) | (0.00034) | (0.00081) | (0.00041) | (0.00007) | (0.04904) |
| L.population density | 0.04810^***^ | 0.03053^***^ | 0.05458^***^ | 0.03634^***^ | 0.00681^***^ | 4.14287^***^ |
|  | (0.00977) | (0.00739) | (0.01840) | (0.00826) | (0.00164) | (0.51565) |
| L.spatial lag population density | 0.02078^***^ | 0.01863^***^ | 0.05261^***^ | 0.01879^***^ | 0.00186^*^ | -0.30937 |
|  | (0.00645) | (0.00415) | (0.01133) | (0.00545) | (0.00100) | (0.52139) |
| L.Share construction of buildings | -0.00003 | -0.00020 | 0.00051 | -0.00007 | 0.00005 | -0.02791 |
|  | (0.00050) | (0.00036) | (0.00099) | (0.00046) | (0.00008) | (0.05071) |
| L.Share specialised construction activities | 0.00400^***^ | 0.00196^**^ | 0.00791^***^ | 0.00327^**^ | 0.00037^*^ | 0.15467 |
|  | (0.00141) | (0.00078) | (0.00284) | (0.00127) | (0.00020) | (0.10025) |
| L.Share land transport | 0.00082 | 0.00053 | 0.00228^*^ | 0.00090^*^ | -0.00002 | -0.00448 |
|  | (0.00055) | (0.00044) | (0.00126) | (0.00048) | (0.00008) | (0.04992) |
| L.Share warehousing, support activities for transport | -0.00034 | -0.00012 | -0.00063 | -0.00020 | -0.00011 | -0.06713^*^ |
|  | (0.00046) | (0.00030) | (0.00093) | (0.00040) | (0.00007) | (0.03627) |
| L.Share accommodation | -0.00129^**^ | -0.00060 | -0.00241^*^ | -0.00097^*^ | -0.00033^***^ | -0.08765 |
|  | (0.00066) | (0.00046) | (0.00135) | (0.00058) | (0.00011) | (0.05701) |
| L.Share food & beverage service activities | -0.00062 | -0.00065 | -0.00118 | -0.00041 | -0.00004 | -0.19721^***^ |
|  | (0.00127) | (0.00069) | (0.00276) | (0.00115) | (0.00016) | (0.07271) |
| L.Share other professional, scientific & technical activities | -0.00040^**^ | -0.00021^*^ | -0.00062 | -0.00036^*^ | -0.00004 | -0.01652 |
|  | (0.00020) | (0.00011) | (0.00042) | (0.00018) | (0.00003) | (0.01544) |
| L.Share temporary employment agency | 0.00039^***^ | 0.00031^**^ | 0.00076^***^ | 0.00033^***^ | 0.00007^***^ | 0.02151 |
|  | (0.00015) | (0.00014) | (0.00026) | (0.00012) | (0.00002) | (0.01499) |
| L.Share services to buildings and landscape activities | 0.00078 | 0.00040 | 0.00075 | 0.00081 | 0.00004 | 0.02670 |
|  | (0.00057) | (0.00034) | (0.00109) | (0.00049) | (0.00008) | (0.04300) |
| L.Share human health activities | -0.00203 | -0.00245^*^ | -0.00217 | -0.00188 | -0.00007 | -0.43636^***^ |
|  | (0.00234) | (0.00137) | (0.00435) | (0.00199) | (0.00038) | (0.12490) |
| L.Share foreign population | 0.00324^***^ | 0.00232^***^ | 0.00803^***^ | 0.00313^***^ | 0.00010 | -0.03685 |
|  | (0.00077) | (0.00055) | (0.00162) | (0.00067) | (0.00012) | (0.06594) |
| L.land price | 0.00033 | 0.00004 | 0.00005 | 0.00032 | 0.00009 | -0.02984 |
|  | (0.00035) | (0.00025) | (0.00071) | (0.00031) | (0.00007) | (0.03773) |
| L.crime rate | 0.00160^***^ | 0.00069^*^ | 0.00144 | 0.00145^***^ | 0.00030^***^ | 0.00984 |
|  | (0.00057) | (0.00041) | (0.00104) | (0.00048) | (0.00010) | (0.05438) |
| L.overnight stays | 0.00235^***^ | 0.00129^**^ | 0.00320^**^ | 0.00223^***^ | 0.00031^***^ | 0.13862^**^ |
|  | (0.00071) | (0.00054) | (0.00144) | (0.00061) | (0.00011) | (0.06612) |
| L.voter turnout | -0.01872^***^ | -0.00897^***^ | -0.01970^***^ | -0.01410^***^ | -0.00234^***^ | -0.54730 |
|  | (0.00352) | (0.00265) | (0.00686) | (0.00296) | (0.00058) | (0.39573) |
| L.flat size | 0.00255 | 0.00987^**^ | 0.01191 | 0.00084 | -0.00073 | 2.36321^***^ |
|  | (0.00693) | (0.00491) | (0.01280) | (0.00590) | (0.00111) | (0.43275) |
| L.recreation area | -0.00319^***^ | -0.00194^***^ | -0.00431^***^ | -0.00279^***^ | -0.00041^***^ | -0.20485^***^ |
|  | (0.00071) | (0.00055) | (0.00135) | (0.00060) | (0.00013) | (0.06187) |
| L.Share creative economy | 0.00008 | -0.00094 | 0.00081 | 0.00028 | -0.00004 | -0.12551^**^ |
|  | (0.00102) | (0.00060) | (0.00243) | (0.00094) | (0.00013) | (0.06300) |
| L.social welfare rate | 0.00024 | 0.00019 | 0.00031 | 0.00021 | 0.00001 | 0.02693^*^ |
|  | (0.00018) | (0.00013) | (0.00035) | (0.00015) | (0.00003) | (0.01545) |
| L.public financial capacity | -0.00065 | -0.00083 | -0.00287 | -0.00024 | 0.00002 | -0.16098 |
| Time effects (reference = 2014) | (0.00098) | (0.00072) | (0.00195) | (0.00083) | (0.00015) | (0.10324) |
| 2006 | -0.00538^***^ | -0.00340^***^ | -0.00840^***^ | -0.00444^***^ | -0.00066^***^ | -0.23317^***^ |
|  | (0.00027) | (0.00019) | (0.00054) | (0.00024) | (0.00004) | (0.03286) |
| 2010 | -0.00476^***^ | -0.00300^***^ | -0.00754^***^ | -0.00393^***^ | -0.00060^***^ | -0.15197^***^ |
|  | (0.00022) | (0.00015) | (0.00044) | (0.00020) | (0.00004) | (0.02380) |
| N | 1,050 | 1,050 | 1,050 | 1,050 | 1,050 | 1,050 |
| R^2^ | 0.69251 | 0.59806 | 0.57814 | 0.67466 | 0.57996 | 0.47040 |
| F-Test | 46.12668 | 32.95818 | 29.31205 | 41.86622 | 22.71191 | 22.06233 |

All models include region-fixed effects. Robust standard errors in parentheses are clustered at the region level, * p < 0.10, ** p < 0.05, *** p < 0.01^.^

**Interpretation of control variables**

When interpreting the effects of the control variables it is important to keep in mind that the effects are identified using the within variation, i.e. the variation of the variables over time only. The cross-sectional variation between regions cannot be used for identification in the fixed effects models. It is not possible to identify effects of time-invariant regional characteristics and effects of slowly changing variables might be weakly identified (see Hausman and Taylor, 1981).

General labor market conditions such as the wage level and regional employment growth seem to be of minor importance for immigrants when choosing a first residence in Germany. The coefficients of these variables do not differ from zero at conventional levels of significance and the positive correlation between the regional unemployment rate and immigration is counterintuitive when considering arguments of migration theory. This applies to the overall immigration rate as well as the models for different sub-groups. The results for the regional labor market conditions are partly in line with evidence provided by Heider et al. (2020) who report a significant positive correlation between regional unemployment and immigration from Eastern European countries.

The type of jobs that are available in the local labor market seem to be more important for workers from abroad who decide about their first workplace and residence in Germany. The immigration rate increases with the local employment share of specialized construction activities and of temporary employment agencies, while regional labor markets specialized in professional, scientific and technical activities and accommodation do not seem to be preferred regions of destination. For employment in land transport we detect a statistically significant effect for the workers with no formal qualification or unknown qualification only.

There is, moreover, a robust positive effect of the share of the foreign population on the overall immigration rate and all sub-groups except for the Non-EU migrants. This outcome confirms results by Tanis (2020) who argues that this finding might point to network effects or the impact of migration-specific amenities. The highly significant positive coefficients of population density and its spatial lag indicate that all groups of international migrants considered here are attracted by large cities and locations close to large cities in Germany. This is in line with similar evidence provided by Heider et al. (2020). The findings for other amenities and disamenties are, in contrast, mixed. While we detect a robust positive effect of overnight stays as a measure of general attractiveness of the region which is in line with theoretical arguments, estimates for the crime rate and the availability of recreational areas are at odds with theoretical expectations.

**Literature**

Hausman J, Taylor W. Panel data and unobservable individual effects. Econometrica. 1981;49: 1377–1398.

Heider B, Stroms P, Koch J, Siedentop S. Where do immigrants move in Germany? The role of international migration in regional disparities in population development. Population, Space and Place. 2020;26(8):1-19. DOI: 10.1002/psp.2363.

Tanis K. Regional Distribution and Location Choices of Immigrants in Germany. Regional Studies. 2020;54(4):483-94. DOI: 10.1080/00343404.2018.1490015.
