# Supplementary material for: Do xenophobic attitudes influence migrant workers’ regional location choice?
Source: PLoS One. 2025 Feb 5;20(2):e0316627. doi: 10.1371/journal.pone.0316627 (PMC11798449; doi:10.1371/journal.pone.0316627)
Supplement: S4 Table — (DOCX) [file pone.0316627.s004.docx]

**S4 Table A4: Instrument variable results for xenophobic violence - controls and first stage**

|  | All | Skilled | Unskilled/unknown qualification | EU | Non-EU | First stage |
| --- | --- | --- | --- | --- | --- | --- |
| Rate xenophobic violence | -0.00144 | -0.00136^**^ | -0.00102 | -0.00142 | -0.00004 |  |
|  | (0.00107) | (0.00067) | (0.00203) | (0.00087) | (0.00030) |  |
| IV supply vocational training |  |  |  |  |  | -0.61676^**^ |
|  |  |  |  |  |  | (0.26729) |
| IV foreign population |  |  |  |  |  | 0.05863^***^ |
|  |  |  |  |  |  | (0.01683) |
| L.unemployment rate | 0.00305^***^ | 0.00076 | 0.00450^**^ | 0.00301^***^ | 0.00014 | 0.51621^***^ |
|  | (0.00085) | (0.00050) | (0.00177) | (0.00074) | (0.00020) | (0.12541) |
| L.wage level | -0.00492^**^ | -0.00517^***^ | -0.01362^***^ | -0.00393^**^ | -0.00103 | 0.41250 |
|  | (0.00212) | (0.00137) | (0.00393) | (0.00192) | (0.00063) | (0.44622) |
| L.employment growth | -0.00026^*^ | 0.00011 | 0.00006 | -0.00002 | 0.00009 | -0.08223^**^ |
|  | (0.00014) | (0.00013) | (0.00037) | (0.00014) | (0.00007) | (0.03217) |
| L.population density | 0.00697 | 0.00164 | -0.01128 | 0.00262 | 0.00428^***^ | 0.22318 |
|  | (0.00443) | (0.00240) | (0.00868) | (0.00367) | (0.00130) | (0.80142) |
| L.spatial lag population density | 0.01152^**^ | 0.00462 | 0.04727^***^ | 0.00865^*^ | 0.00244 | -1.98744^**^ |
|  | (0.00577) | (0.00335) | (0.01205) | (0.00495) | (0.00153) | (0.79022) |
| L.Share construction of buildings | 0.00053 | 0.00019 | 0.00065 | 0.00037 | 0.00019^*^ | 0.00774 |
|  | (0.00038) | (0.00021) | (0.00088) | (0.00033) | (0.00010) | (0.06300) |
| L.Share specialised construction activities | 0.00082 | -0.00001 | 0.00494^**^ | 0.00059 | 0.00040^*^ | -0.45284^**^ |
|  | (0.00101) | (0.00055) | (0.00231) | (0.00089) | (0.00023) | (0.17899) |
| L.Share land transport | -0.00013 | -0.00012 | 0.00005 | 0.00015 | -0.00015^*^ | -0.04408 |
|  | (0.00025) | (0.00021) | (0.00071) | (0.00024) | (0.00008) | (0.06941) |
| L.Share warehousing, support activities for transport | -0.00035 | -0.00023 | -0.00054 | -0.00014 | -0.00016^*^ | -0.01793 |
|  | (0.00039) | (0.00018) | (0.00087) | (0.00033) | (0.00009) | (0.05223) |
| L.Share accommodation | -0.00047 | -0.00076^***^ | -0.00048 | -0.00050 | -0.00012 | -0.16234^**^ |
|  | (0.00034) | (0.00028) | (0.00079) | (0.00030) | (0.00011) | (0.07597) |
| L.Share food & beverage service activities | 0.00070 | 0.00020 | 0.00301 | 0.00072 | 0.00001 | 0.03488 |
|  | (0.00121) | (0.00063) | (0.00258) | (0.00103) | (0.00027) | (0.12505) |
| L.Share other professional, scientific & technical activities | -0.00009 | -0.00002 | 0.00014 | -0.00009 | -0.00001 | -0.01932 |
|  | (0.00017) | (0.00012) | (0.00031) | (0.00015) | (0.00004) | (0.02426) |
| L.Share temporary employment agency | 0.00008 | -0.00007 | 0.00025 | 0.00004 | 0.00001 | 0.04930 |
|  | (0.00012) | (0.00009) | (0.00027) | (0.00011) | (0.00003) | (0.03376) |
| L.Share services to buildings and landscape activities | 0.00051 | 0.00015 | 0.00094 | 0.00025 | 0.00024^**^ | 0.06987 |
|  | (0.00032) | (0.00024) | (0.00082) | (0.00027) | (0.00010) | (0.06776) |
| L.Share human health activities | 0.00091 | 0.00150^**^ | 0.00137 | 0.00093 | -0.00015 | 0.38477^**^ |
|  | (0.00092) | (0.00063) | (0.00213) | (0.00075) | (0.00036) | (0.19370) |
| L.Share foreign population | 0.00138^**^ | 0.00118^***^ | 0.00341^***^ | 0.00163^***^ | -0.00035^***^ | 0.00453 |
|  | (0.00054) | (0.00027) | (0.00121) | (0.00048) | (0.00010) | (0.06578) |
| L.land price | 0.00013 | 0.00008 | 0.00036 | 0.00015 | 0.00003 | 0.07473^*^ |
|  | (0.00014) | (0.00012) | (0.00028) | (0.00013) | (0.00005) | (0.04322) |
| L.crime rate | 0.00040 | 0.00014 | -0.00053 | 0.00011 | 0.00025^**^ | 0.15569^*^ |
|  | (0.00035) | (0.00025) | (0.00088) | (0.00033) | (0.00011) | (0.08859) |
| L.overnight stays | 0.00028 | 0.00043 | -0.00265^*^ | 0.00031 | 0.00011 | 0.29168^***^ |
|  | (0.00076) | (0.00041) | (0.00145) | (0.00065) | (0.00018) | (0.09548) |
| L.voter turnout | -0.00457^**^ | 0.00279^**^ | -0.01438^***^ | -0.00272^*^ | -0.00071 | 0.24419 |
|  | (0.00179) | (0.00129) | (0.00464) | (0.00158) | (0.00051) | (0.48690) |
| L.flat size | -0.01826^***^ | -0.00519^**^ | -0.02086^**^ | -0.01309^***^ | -0.00509^***^ | 0.77029 |
|  | (0.00423) | (0.00219) | (0.00899) | (0.00379) | (0.00077) | (0.59448) |
| L.recreation area | 0.00016 | 0.00054 | -0.00035 | 0.00028 | -0.00020 | 0.30738^***^ |
|  | (0.00055) | (0.00036) | (0.00129) | (0.00048) | (0.00013) | (0.08130) |
| L.Share creative economy | 0.00020 | -0.00057^*^ | 0.00115 | 0.00038 | -0.00009 | 0.09776 |
|  | (0.00061) | (0.00030) | (0.00114) | (0.00052) | (0.00015) | (0.07812) |
| L.Social welfare rate | 0.00029^*^ | 0.00013 | 0.00048^*^ | 0.00021^*^ | 0.00008^*^ | 0.03503 |
|  | (0.00015) | (0.00009) | (0.00029) | (0.00012) | (0.00004) | (0.03272) |
| L.Public financial capacity | -0.00078 | -0.00061^*^ | -0.00028 | -0.00052 | -0.00027^**^ | 0.00284 |
|  | (0.00051) | (0.00033) | (0.00107) | (0.00048) | (0.00012) | (0.08637) |
| Time effects (reference = 2006) |  |  |  |  |  |  |
| 2004 | 0.00008 | 0.00026 | 0.00007 | 0.00013 | -0.00004 | 0.22145^***^ |
|  | (0.00026) | (0.00017) | (0.00053) | (0.00021) | (0.00009) | (0.05922) |
| 2005 | -0.00028^*^ | 0.00002 | -0.00056^*^ | -0.00009 | -0.00017^***^ | 0.10671^**^ |
|  | (0.00016) | (0.00011) | (0.00032) | (0.00014) | (0.00005) | (0.04397) |
| 2007 | -0.00026 | -0.00044 | 0.00043 | -0.00053 | 0.00027^*^ | -0.53477^***^ |
|  | (0.00058) | (0.00037) | (0.00113) | (0.00049) | (0.00016) | (0.04614) |
| 2008 | -0.00033 | -0.00050 | 0.00008 | -0.00051 | 0.00016 | -0.62850^***^ |
|  | (0.00070) | (0.00044) | (0.00132) | (0.00058) | (0.00020) | (0.04739) |
| 2009 | -0.00057 | -0.00063 | -0.00052 | -0.00053 | -0.00005 | -0.65686^***^ |
|  | (0.00075) | (0.00046) | (0.00141) | (0.00062) | (0.00021) | (0.05589) |
| 2010 | -0.00039 | -0.00052 | -0.00022 | -0.00045 | 0.00006 | -0.77745^***^ |
|  | (0.00088) | (0.00055) | (0.00166) | (0.00073) | (0.00024) | (0.05249) |
| 2011 | 0.00028 | -0.00044 | 0.00204 | 0.00004 | 0.00027 | -1.18796^***^ |
|  | (0.00132) | (0.00082) | (0.00252) | (0.00110) | (0.00036) | (0.05545) |
| 2012 | 0.00080 | 0.00111 | 0.00005 | 0.00054 | 0.00028 | -1.27541^***^ |
|  | (0.00144) | (0.00090) | (0.00274) | (0.00118) | (0.00039) | (0.05397) |
| 2013 | 0.00097 | 0.00021 | 0.00190 | 0.00063 | 0.00034 | -1.28834^***^ |
|  | (0.00145) | (0.00090) | (0.00278) | (0.00120) | (0.00040) | (0.05378) |
| 2014 | 0.00243 | 0.00080 | 0.00510^*^ | 0.00177 | 0.00058 | -1.30570^***^ |
|  | (0.00149) | (0.00092) | (0.00286) | (0.00123) | (0.00040) | (0.05403) |
| 2015 | 0.00284^*^ | 0.00079 | 0.00622^**^ | 0.00172 | 0.00099^**^ | -1.38602^***^ |
|  | (0.00157) | (0.00097) | (0.00303) | (0.00129) | (0.00042) | (0.05563) |
| 2016 | 0.00307^**^ | 0.00056 | 0.00737^**^ | 0.00138 | 0.00159^***^ | -1.37687^***^ |
|  | (0.00155) | (0.00096) | (0.00300) | (0.00128) | (0.00042) | (0.05685) |
| 2017 | 0.00336^**^ | 0.00041 | 0.00860^***^ | 0.00118 | 0.00210^***^ | -1.36059^***^ |
|  | (0.00155) | (0.00095) | (0.00296) | (0.00127) | (0.00042) | (0.05751) |
| N | 2,772 | 2,772 | 2,772 | 2,772 | 2,772 | 2,772 |
| R^2^ | 0.71863 | 0.48365 | 0.69164 | 0.58093 | 0.80077 | 0.56710 |
| F-Test | 63.11184 | 30.92394 | 58.08999 | 29.94332 | 97.43942 | 120.90418 |

All models include region-fixed effects to control for unobserved time-constant factors. Robust standard errors in parentheses are clustered at the region level,

* p < 0.10, ** p < 0.05, *** p < 0.01.
