# Supplementary material for: Do xenophobic attitudes influence migrant workers’ regional location choice?
Source: PLoS One. 2025 Feb 5;20(2):e0316627. doi: 10.1371/journal.pone.0316627 (PMC11798449; doi:10.1371/journal.pone.0316627)
Supplement: S5 Table — (DOCX) [file pone.0316627.s005.docx]

**S5 Instrument discussion, Table 5a, Table 5b**

**Instrument variable estimation – validity of instruments**

To be valid, the instruments must be relevant and exogenous. They should correlate with xenophobic attitudes, must be uncorrelated with the error term $\varepsilon_{it}$ in the migration models (2) and (3), and should influence the immigration rate only through their effect on xenophobic attitudes. Apart from the discussion of theoretical arguments in Section 4, we provide evidence based on different standard econometric tests (First-stage results of the instruments, F-Test of excluded instruments, Kleibergen-Paap LM test, Anderson-Rubin test) to check whether our assumptions regarding validity of the instruments likely hold. In particular, we apply the Hansen J-Test to examine whether the assumption that instruments are exogenous is possibly met. To make use of this test we need at least two instruments for each of the two xenophobia measures. All results of these tests are displayed in the Table 1, 2, and 3 in the main text. Finally, we estimate reduced form regression to further test the intuition behind our instruments (see Table A5a and A5b). However, in the end, it is not possible to **prove** that the exclusion restrictions of the instrument variables hold.

Applying a fixed effect instrumental variable estimation, we require time-varying instruments. There is annual information available for the first three instrument variables (local supply of vocational training, share of low-skilled foreign workers, percentage of foreign population). For a treatment that varies with the location of the region, we interact our fourth instrument, the longitude of the county centre, with the time-varying share of low-skilled foreign workers and the percentage of the foreign population. Thus, changes in the instruments over time are used to explain changes in xenophobic attitudes in the first-stage regressions.

**Relevance and exogeneity of instruments: first-stage results and tests on relevance and exogeneity**

The lower panel of Table 1 summarizes the results of the IV estimations for the model including right-wing votes. Table A3 in the Supporting Information 3 shows results of the complete first-stage regression. The different tests on validity of the instruments (local supply of vocational training, share of low-skilled foreign workers interacted with longitude) indicate that we cannot reject that they are exogenous and relevant. The Hansen J-statistic suggests that we cannot reject the hypothesis that the two instruments are exogenous (p-values > 0.10). The first-stage results indicate that the instruments are relevant because the coefficient estimates of the instruments are statistically significant (local supply of vocational training at the 5%-level, share of low-skilled foreign workers interacted with longitude at the 1%-level) and the signs are in line with theoretical expectations discussed in Section 4: a declining supply of vocational training positions is associated with increasing support for right-wing parties. The positive correlation between the share of low-skilled foreign workers and the votes of right-wing parties is consistent with the labour market competition argument outlined in Section 4. Moreover, according to the Kleibergen-Paap LM tests, our instruments should be adequate for the identification of the model (p-value < 0.05), which is confirmed by the F-statistics of excluded instruments (> 10) and the Anderson-Rubin test of the significance of our pivotal variable, right-wing votes (p-value < 0.05). Finally, results of reduced form regressions are in line with the intuition behind our instruments (see Table A5a below along with detailed discussion).

The lower panel of Table 2 summarizes the results of the IV estimations for the model including xenophobic violence. Table A4 in the Supporting Information 4 shows results of the complete first-stage regression. The different tests on validity of the instruments (local supply of vocational training, share of foreign population interacted with longitude) indicate that we cannot reject that they are exogenous and relevant. The Hansen J-statistic suggests that we cannot reject the hypothesis that the two instruments (local supply of vocational training, share of low-skilled foreign workers interacted with longitude) are exogenous (p-values > 0.10). The first-stage results indicate that the instruments are relevant. The coefficients of the instruments are statistically significant (local supply of vocational training at the 5%-level, share of foreign population interacted with longitude at the 1%-level) and the signs are in line with theoretical expectations discussed in Section 4: an increasing supply of vocational training positions is associated with a decline of xenophobic violence. The positive correlation between the share of the foreign population and the number of xenophobic crimes per worker is consistent with the ‘racial threat hypothesis’ outlined in Section 3. Moreover, according to the Kleibergen-Paap LM tests, our instruments should be adequate for the identification of the model (p-value < 0.05), which is confirmed by the F-statistics of excluded instruments (> 10). However, the Anderson-Rubin test of the significance of our pivotal variable, xenophobic violence, indicates that the effect of xenophobic attitudes does not statistically significantly differ from zero in most models (p-value partly > 0.05). We detect an important effect only for the skilled immigrants. Finally, results of reduced form regressions are in line with the intuition behind our instruments (see Table A5b below and the discussion).

**Reduced form regressions**

Following Levitt (2002) we use reduced form regressions with the instrumental variables as explanatory variables in the main model excluding the endogenous variable of interest, i.e. the indicator of xenophobic attitudes in our case, to further test the intuition behind our instruments. When using these reduced form regressions to check the validity of an instrumental variable, it would pose a problem to find that the coefficient of an instrumental variable has a sign that is at odds with the instrument’s story, as argued by Murray (2006). The results of these regressions are summarized in Table A5a (instruments for right-wing votes) and Table A5b (instruments for the rate of xenophobic violence). They are in line with the intuition behind our instruments.

**S5_Table A5a:** **Instrument validity for right-wing votes - reduced form regressions**

|  | All immigrants | Skilled | Unskilled/Unknown Qualification | EU | Non-EU |
| --- | --- | --- | --- | --- | --- |
| IV supply vocational training | 0.00004^**^ | 0.00002^**^ | 0.00007^*^ | 0.00003 | 0.00001^***^ |
|  | (0.00002) | (0.00001) | (0.00004) | (0.00002) | (0.00000) |
| IV low-skilled foreign workers | -0.00049^***^ | -0.00040^***^ | -0.00082^***^ | -0.00038^***^ | -0.00003 |
|  | (0.00013) | (0.00009) | (0.00026) | (0.00012) | (0.00002) |
| Share of foreign population | 0.00354^***^ | 0.00256^***^ | 0.00853^***^ | 0.00336^***^ | 0.00013 |
|  | (0.00059) | (0.00037) | (0.00138) | (0.00055) | (0.00010) |
| N | 1,052 | 1,052 | 1,052 | 1,052 | 1,052 |
| R^2^ | 0.82850 | 0.82180 | 0.71270 | 0.78942 | 0.66884 |
| R^2^ overall | 0.10793 | 0.05468 | 0.05225 | 0.05951 | 0.37234 |
| R^2^ within | 0.82850 | 0.82180 | 0.71270 | 0.78942 | 0.66884 |
| R^2^ between | 0.21689 | 0.10858 | 0.09048 | 0.13150 | 0.42847 |

Notes: Regression results for main model excluding the share of right-wing votes and including instead the two instruments for the share of right-wing votes.

All models include time-varying explanatory variables and region- as well as time-fixed effects to control for observed and unobserved factors.

Robust standard errors in parentheses are clustered at the region level, * p < 0.10, ** p < 0.05, *** p < 0.01.

IV supply vocational training: lagged (1 year) ratio of vocational training positions to graduates demanding training. IV low-skilled foreign workers:

lagged (2 years) share of low-skilled among foreign workers interacted with longitude.

The regional supply of vocational training (ratio of vocational training positions to graduates demanding training) is positively correlated with immigration rates in Table A5a. Under the assumption that exclusion of the supply of vocational training from the immigration model is valid, the combination of a negative first-stage coefficient of vocational training supply (see Table 1 and Table A3) and a positive reduced form coefficient implies that the instrumental variables estimate of xenophobic attitudes will be negative. This also applies to the second instrument, the share of low-skilled among foreign workers interacted with longitude, where the combination of a positive first-stage coefficient and a negative reduced form coefficient is in line with a negative instrumental variable estimate for the share of right-wing votes. With respect to the latter instrument, it is important to keep in mind that we include the regional share of the foreign population as control variable in the main equation of the IV estimation and also in the reduced form model (see corresponding results in Table A3 and A5a).

**S5_Table A5b: Instrument validity for rate of xenophobic violence - reduced form regressions**

|  | All immigrants | Skilled | Unskilled/Unknown Qualification | EU | Non-EU |
| --- | --- | --- | --- | --- | --- |
| IV supply vocational training | 0.00176 | 0.00008 | 0.00575^*^ | 0.00160 | -0.00037 |
|  | (0.00136) | (0.00071) | (0.00295) | (0.00116) | (0.00039) |
| IV foreign population | -0.00007 | -0.00009^**^ | 0.00003 | -0.00007 | -0.00001 |
|  | (0.00007) | (0.00004) | (0.00015) | (0.00006) | (0.00002) |
| Share of foreign population | 0.00138^**^ | 0.00117^***^ | 0.00346^***^ | 0.00163^***^ | -0.00036^***^ |
|  | (0.00054) | (0.00026) | (0.00122) | (0.00047) | (0.00011) |
| N | 2,778 | 2,778 | 2,778 | 2,778 | 2,778 |
| R^2^ | 0.76469 | 0.60725 | 0.69550 | 0.65538 | 0.80146 |
| R^2^ overall | 0.05208 | 0.01697 | 0.02086 | 0.01984 | 0.24298 |
| R^2^ within | 0.76469 | 0.60725 | 0.69550 | 0.65538 | 0.80146 |
| R^2^ between | 0.04255 | 0.00906 | 0.02049 | 0.01062 | 0.26561 |

Notes: Regression results for main model excluding the rate of xenophobic violence and including instead the two instruments for the rate of xenophobic violence.

All models include time-varying explanatory variables and region- as well as time-fixed effects to control for observed and unobserved factors.

Robust standard errors in parentheses are clustered at the region level; * p < 0.10, ** p < 0.05, *** p < 0.01.

IV supply vocational training: lagged (1 year) log ratio of vocational training positions to graduates demanding training. IV foreign population:

lagged (9 years) log share of foreign population interacted with longitude.

The regional supply of vocational training (ratio of vocational training positions to graduates demanding training) is also positively correlated with the immigration rates in Table A5b. Assuming again that exclusion of the supply of vocational training from the immigration model is valid, the combination of a negative first-stage coefficient of vocational training supply (see Table 2 and Table S4_A4) and a positive reduced form coefficient implies that the instrumental variables estimate of xenophobic behavior will be negative. The second instrument is the share of the foreign population lagged by 9 years interacted with longitude. For this instrument it is in particular important to notice that we include the share of the foreign population lagged by one year as an explanatory variable in the immigration model. In the fixed effects IV models, only the variation over time is used in the estimation procedure. The correlation between the first differences of the two variables (IV foreign population, contemporaneous share foreign population) is close to zero (correlation coefficient: -0.0381), while there is a strong positive correlation of the levels (correlation coefficient: 0.8915). This implies that regions which experienced a relatively strong increase in the share of the foreign population in the period under consideration did not necessarily experience a strong increase 9 years before. Thus, the variable lagged by 9 years cannot be used as a proxy for the share of the foreign population lagged by one year, i.e. the instrument does not capture the effects of the contemporaneous share of the foreign population. The reduced form coefficients of the IV foreign population are negative in Table A5b, the only exception being the model for the unskilled immigrants/those with unknown qualification. Thus, the correlation between the share of the foreign population lagged by 9 years and the immigration rate tends to be negative in reduced models. A positive first-stage coefficient of the IV foreign population in Table 2 combined with the negative reduced form coefficient in Table 5b is in line with a negative instrumental variable estimate for the rate of xenophobic violence.

**Literature**

Levitt, Steven D. Using Electoral Cycles in Police Hiring to Estimate the Effect of Police on Crime: Reply. American Economic Review. 2002;92 (4):1244–50. DOI: 10.1257/00028280260344777.

Murray, Michael, P. Avoiding Invalid Instruments and Coping with Weak Instruments. Journal of Economic Perspectives. 2006;20(4):111-132. DOI: 10.1257/jep.20.4.111.
