# Supplementary material for: Do xenophobic attitudes influence migrant workers’ regional location choice?
Source: PLoS One. 2025 Feb 5;20(2):e0316627. doi: 10.1371/journal.pone.0316627 (PMC11798449; doi:10.1371/journal.pone.0316627)
Supplement: S6 Table — (DOCX) [file pone.0316627.s006.docx]

**S6 Table A6: Unconditional correlations between immigration rate, share of right-wing votes and rate of xenophobic violence**

|  | (1) | (2) |
| --- | --- | --- |
| Share right-wing votes | -0.00104^***^ |  |
|  | (0.00012) |  |
| Rate xenophobic violence |  | -0.00151^***^ |
|  |  | (0.00006) |
| N | 1,052 | 2,778 |
| R^2^ | 0.43 | 0.43 |

*Standard errors in parentheses: ^*^ p < 0.10, ^**^ p < 0.05, ^***^ p < 0.01*

*Notes: All models include time-fixed effects.*
