# Supplementary material for: Do xenophobic attitudes influence migrant workers’ regional location choice?
Source: PLoS One. 2025 Feb 5;20(2):e0316627. doi: 10.1371/journal.pone.0316627 (PMC11798449; doi:10.1371/journal.pone.0316627)
Supplement: S7 Table — (DOCX) [file pone.0316627.s007.docx]

**S7 Table A7: Fixed effects models with right-wing votes and xenophobic violence**

| Fixed effects models | | | | | |
| --- | --- | --- | --- | --- | --- |
|  | (1)  All immigrants | (2)  Skilled | (3)  Unskilled/unknown qualification | (4)  EU | (5)  Non-EU |
| Share of right-wing votes | -0.00205^**^  (0.00110) | -0.00069  (0.00048) | -0.00353  (0.00220) | -0.00199^**^  (0.00098) | -.00011  (0.00016) |
| Xenophobic violence | -0.00008  (0.00034) | -0.00004  (0.00016) | -0.00057  (0.00080) | -0.00010  (0.00030) | 0.00004  (0.00006) |
| N | 488 | 488 | 488 | 488 | 488 |
| R^2^ overall | 0.05 | 0.02 | 0.02 | 0.03 | 0.25 |
| R^2^ within | 0.77 | 0.82 | 0.71 | 0.76 | 0.72 |
| R^2^ between | 0.02 | 0.00 | 0.01 | 0.01 | 0.21 |

*All models include time-varying explanatory variables and region- as well as time-fixed effects to control for observed and unobserved factors. Robust standard errors in parentheses are clustered at the region level, * p < 0.10, ** p < 0.05, *** p < 0.01.*

To investigate whether xenophobic attacks and the support for right-wing parties have separate effects on the immigration rate, we also estimate fixed effects models that include the two indicators simultaneously. The results for the main variables of interest are summarized in Table A7. However, estimating this model comes at some costs. The sample size strongly declines because there is no perfect overlap of the two indicators for xenophobic attitudes. The number of observations for the model including the right-wing votes only is 1,050. If we add the variable for xenophobic violence the number of observations declines to 488. The reduction of the sample size affects the precision of estimates and as a result standard errors increase notably.
