# Supplementary material for: Do xenophobic attitudes influence migrant workers’ regional location choice?
Source: PLoS One. 2025 Feb 5;20(2):e0316627. doi: 10.1371/journal.pone.0316627 (PMC11798449; doi:10.1371/journal.pone.0316627)
Supplement: S8 Table — (DOCX) [file pone.0316627.s008.docx]

**S8 Table A8: Summary statistics for the share of selected immigrant groups in total immigrant population**

|  | mean | sd | min | max |
| --- | --- | --- | --- | --- |
| Polish | 0.123 | 0.053 | 0.063 | 0.246 |
| Romanian | 0.085 | 0.061 | 0.022 | 0.210 |
| Bulgarian | 0.036 | 0.028 | 0.009 | 0.104 |
| Hungarian | 0.032 | 0.025 | 0.008 | 0.086 |
| Croatian | 0.025 | 0.023 | 0.007 | 0.074 |
| Turkish | 0.056 | 0.033 | 0.017 | 0.100 |
| Italian | 0.035 | 0.007 | 0.027 | 0.054 |
| Greek | 0.025 | 0.010 | 0.013 | 0.044 |
| Russian | 0.032 | 0.017 | 0.012 | 0.060 |
| Indian | 0.019 | 0.004 | 0.010 | 0.029 |
| Spanish | 0.017 | 0.008 | 0.010 | 0.037 |
| Chinese | 0.021 | 0.006 | 0.013 | 0.031 |

The IEB data includes approximately 1,729,970 individual immigration events, i.e. first employment notifications of foreign workers in the IEB between 2004 and 2017 as defined in Section 4 of the manuscript. The 12 largest groups of foreign workers who reside in Germany include Bulgarian, Chinese, Croatian, Greek, Hungarian, Indian, Italian, Polish, Romanian, Russian, Spanish, Syrian, Turkish. They account for almost 59% of all immigration events in our data set. Table A8 shows the mean share of the different immigrant groups in the total immigrant population as well as standard deviation and the minimum and maximum share for the period 2004 to 2017. The average share of the largest groups varies between 12.3% for the Polish immigrants and 1.7% for immigrants from Spain. However, the range is much higher for specific years as indicated by the minimum and maximum percentages.
