# Supplementary material for: Do xenophobic attitudes influence migrant workers’ regional location choice?
Source: PLoS One. 2025 Feb 5;20(2):e0316627. doi: 10.1371/journal.pone.0316627 (PMC11798449; doi:10.1371/journal.pone.0316627)
Supplement: Attachment — Submitted filename: 241101_Response letter.docx [file pone.0316627.s009.docx]

1^st^ November, 2024

Dear Professor Erisen and dear referees,

we would like to thank you very much for the helpful and constructive comments on our manuscript *Does xenophobic behaviour influence migrant workers´ regional location choice?* We are grateful for the opportunity to revise and resubmit our paper and appreciate the detailed suggestions. We think that we were able to deal with the issues that were raised by you and have marked the fundamentally revised and added parts with tracked changes. In the attached document, we respond to your comments and describe in detail how we dealt with them. We first repeat each comment in italic, black script and then answer in blue script.

Best wishes,

Carola Burkert, Tanja Buch, Stefan Hell, Annekatrin Niebuhr, Anette Haas

**Replay to the Editor**

1) *Need for a clearer definition and theoretical discussion of the main concepts (e.g., xenophobic behavior and attitude) early in the introduction.*

So far, we have used the terms xenophobic attitudes and xenophobic behavior interchangeably. After a critical discussion, we believe that “xenophobic attitudes” is the umbrella term that includes “xenophobic behavior”. In literature, both voting behavior and xenophobic violence are used to investigate the development and effects of xenophobic attitudes (e.g. Entorf and Lange 2023, Falk et al. 2011). We decided to use the term xenophobic attitudes strictly to prevent misunderstandings. With this in mind, we also changed the title of the paper. We thank you very much for pointing out our inaccuracy.

We added some sentences to the introduction in which we argue, with reference to the literature, that votes for right-wing parties and xenophobic violence can be used as proxies for right-wing attitudes:

“This might affect regions very differently because stereotypes and prejudices have a strong local dimension, as they contribute to the community culture, which defines the broader societal traits and relations that shape places in terms of the prevailing mindset and overall way of life (Denti et al. 2023, Zajak et al. 2024). […] This paper investigates the effects of regional differences in xenophobic attitudes, measured by votes for right-wing parties in federal elections and xenophobic violence, on the immigration of foreign workers to German district regions. The electoral success of right-wing parties is used as a proxy for xenophobic attitudes, as there is evidence that anti-immigration sentiments are highly correlated with right-wing party support (Arzheimer and Berning 2019) and that the results of national elections reflect local values and attitudes (Bellodi et al. 2024). Our data on xenophobic violence should also indicate the extent of xenophobic attitudes in a region as it is based on regional information from the German security authorities, which records crimes with racist, xenophobic or anti-Semitic motives.”

2) *More explicit statement of hypotheses and their alignment with the literature.*

We have now explicitly named the hypotheses that we derived from the literature (H1-H4a/b) and strengthened the theoretical discussion around the hypotheses in section 3. In section 5, we added Table 4 that provides an overview on whether the hypotheses are confirmed by the regression results or not. In section 6, when discussing the results, we explicitly state whether the hypothesis under discussion is confirmed or not.

3) *In addition, expanding the literature review, by involving political science research, particularly on the subject of immigration attitudes and far-right vote choice in Germany.*

We extended the literature review, inter alia by involving political science research, as follows:

Line 46ff (Version with track changes):
This might affect regions very differently because stereotypes and prejudices have a strong local dimension, as they contribute to the community culture, which defines the broader societal traits and relations that shape places in terms of the prevailing mindset and overall way of life (Denti et al. 2023, Zajak et al. 2024).

Line 61ff:
The electoral success of right-wing parties is used as a proxy for xenophobic attitudes as there is evidence that anti-immigration sentiments are highly correlated with right-wing party support (e.g., Arzheimer and Berning 2019) and that the results of national elections reflect local values and attitudes (Bellodi et al. 2024).

Line 154ff:

For a discussion of the reasons why right-wing extremist attitudes find strong support especially in East Germany, see, e.g., Ireland (1997) and Weisskircher (2020).

Line 228ff:

Skilled people seem to be more sensitive to xenophobic behaviour attitudes (Knabe et al. 2013). This could be due to a stronger intention of better educated immigrants to identify and integrate with their host country, resulting in stronger disappointment if the native-born population reveals negative perceptions of immigration (Knabe et al. 2013). Moreover, skilled people and tend to have more regional job opportunities to choose from (Avidu 2018).

Line 237 ff:

There are differences in the freedom to choose where to live between migrant workers from the EU and those from third countries. Labour migrants from third countries have no free access to the German labour market and in general their residence permit is linked to a labour contract. In contrast, labour migrants from EU-countries face no restrictions in their location choice (full freedom of movement for workers). Third country nationals might therefore be less able to avoid a xenophobic environment than EU-nationals when choosing a region of residence. Additionally, media coverage of social and political phenomena tends to decline with increasing regional distance (Branton and Dunaway 2009; Bendix and Liebler 2004). Thus we might expect that EU foreigners react more sensitively to revealed anti-immigrant attitudes because they are better informed than people from more distant third countries.

Line 260ff:
On the one hand, it is conceivable that migrants are more likely to move to regions with right-wing natives if they assume that a large coethnic group offers mental support (Shankley and Laurence 2022). Showing that the experience of racism is lower in places of higher ethnic density, Bécares et al. (2009) speak of a “buffering effect” of ethnic density.

Line 266ff:
On the other hand, a large coethnic group can collect and spread more information, e.g. via mass media (Fennema 2004). If the information is about the hostile attitudes of the local population and warns against immigration, this may amplify the adverse effects of xenophobic behavior attitudes.

Line 409 ff:

Accordingly, people are less likely to commit right-wing extremist attacks where right-wing parties represent a relevant force within the political system and thus an alternative way of exerting pressure on political elites (Koopmans 1996).
Line 887ff.

This is particularly true given that the AfD's strong electoral successes in the last elections were repeatedly seen as a sign that the right-wing ideologies and extreme positions are increasingly accepted by the middle of society (e.g., Biskamp 2024) – which in turn presumably increases the repellent effect on immigration.

4) *Finally, both reviewers highlight the importance of clearly displaying control variables in the regression models and refining the presentation of the results, including justifying methodological choices such as the use of logs and the inability to analyze right-wing votes and xenophobic violence simultaneously.*

Many thanks for these suggestions. Table S3 A3 and S4 A4 show the results for all control variables of the fixed effects IV estimations. Maybe this was not clearly communicated in the previous version of the paper. We added a reference in the first paragraph of section 5. We prefer to exclude the results for the controls from the main Tables 1 to 3 because the tables already contain a lot of information and might become confusing when 27 more coefficients and standard errors are added for 5 different models. Moreover, we think that discussing all the effects of the control variables and comparing effects across different skill groups will not increase the readability of the paper but distract the reader from the main findings, i.e. how xenophobic attitudes affect the immigration rate. However, we now provide a summary of the main results for the control variables together with Table A3 in the Supporting Information S3.

Furthermore, we extended the discussion of the theoretical arguments behind the instrument variables in section 4 and try to explain the mechanisms that are supposed to be at work in more detail. Moreover, we moved most of the information on the econometric tests applied to investigate the relevance and exogeneity of the instruments from section 4 and section 5 to the Supporting Information and discuss the results there in more detail. We hope that this reduces the complexity of these paragraphs while at the same time strengthening the arguments for the validity of the instruments.

Using logarithms of the dependent and the explanatory variables in the model is a rather common approach in migration research (see e.g. Gorinas and Pytliková 2017, Heider et al., 2020 or Mitze, 2019). This specification has also the advantage that all coefficients can be easily interpreted as elasticities, i.e. they give the percentage change of the immigration rate for a change of the corresponding explanatory variable by 1%. We added this information in section 4.2 below equation (2). However, we refrain from a more detailed discussion of econometric advantages (e.g. measurement errors and outliers become less important).

We added an analysis where right-wing votes and xenophobic violence are both included in the model. We briefly discuss the results in section 5. Table A7 with the results is included in the Supporting Information S7. The results are qualitatively in line with our main results, i.e. the coefficients of the variables are negative with the absolute size being larger and increasing for the right-wing votes. While the adverse effect of the right-wing votes is thus confirmed by this extended model, evidence on an important effect of xenophobic violence is less robust. However, including both variables in one model comes at some costs. The sample size strongly declines because there is no perfect overlap of the two variables. The number of observations for the model including the right-wing votes only is 1,050. If we add the variable for xenophobic violence the number of observations declines to 488. The reduction of the sample size affects the precision of estimates and as a result standard errors increase significantly.

**Reply to Reviewer #1**

We are very grateful for the helpful comments of reviewer 1, which contributed to significantly improve our manuscript. We have taken these remarks into account and have substantially revised our paper. In the following, we describe in detail how we dealt with your comments. We first repeat each comment in italic, black script and then answer in blue script.

1. *The paper could benefit from a clearer definition of "xenophobic behavior" early in the introduction. While it is operationalized through right-wing party votes and xenophobic violence, a theoretical discussion would provide better context.*

So far, we have used the terms xenophobic attitudes and xenophobic behavior interchangeably. After a critical discussion, we believe that “xenophobic attitudes” is the umbrella term that includes “xenophobic behavior”. In literature, both voting behavior and xenophobic violence are used to investigate the development and effects of xenophobic attitudes (e.g. Entorf and Lange 2023, Falk et al. 2011). We decided to use the term xenophobic attitudes strictly to prevent misunderstandings. With this in mind, we also changed the title of the paper. We thank you very much for pointing out our inaccuracy.

We added some sentences to the introduction in which we argue, with reference to the literature, that votes for right-wing parties and xenophobic violence can be used as proxies for right-wing attitudes:

“This might affect regions very differently because stereotypes and prejudices have a strong local dimension, as they contribute to the community culture, which defines the broader societal traits and relations that shape places in terms of the prevailing mindset and overall way of life (Denti et al. 2023, Zajak et al. 2024). […] This paper investigates the effects of regional differences in xenophobic attitudes, measured by votes for right-wing parties in federal elections and xenophobic violence, on the immigration of foreign workers to German district regions. The electoral success of right-wing parties is used as a proxy for xenophobic attitudes, as there is evidence that anti-immigration sentiments are highly correlated with right-wing party support (Arzheimer and Berning 2019) and that the results of national elections reflect local values and attitudes (Bellodi et al. 2024). Our data on xenophobic violence should also indicate the extent of xenophobic attitudes in a region as it is based on regional information from the German security authorities, which records crimes with racist, xenophobic or anti-Semitic motives.”

*2. The clear and directional hypothesis is not explicitly stated in the text. Explicitly stating the hypotheses as separate sentences and* *explaining why these hypotheses are used with references to the literature would strengthen the article. The lack of clearly stated hypotheses creates uncertainty about which hypothesis the concepts in the operationalization section are intended to test. The hypotheses should be clearly written, and it should be explicitly stated which concept measures the variable in each hypothesis.*

We have now explicitly named the hypotheses that we derived from the literature (H1-H4a/b) and strengthened the theoretical discussion around the hypotheses in section 3. In section 5, we added Table 4 that provides an overview on whether the hypotheses are confirmed by the regression results or not. In section 6, when discussing the results, we explicitly state whether the hypothesis under discussion is confirmed or not.

*3. In the literature section, the racial threat hypothesis and contact theory are compared, and it is mentioned that contact theory will be used. However, instead of building the literature review part on contact theory, it predominantly centers on xenophobia and attitudes towards immigrants literature. Since contact theory is used, the literature should be further strengthened with references to how contact, especially intimate contact, affects labor immigrants (both skilled and unskilled) and vice versa—how contact affects the xenophobic attitudes and attitudes towards immigrants of German citizens, as well as the attitudes of labor immigrants regarding their entry into the job market. Furthermore, expand the literature review to include more “contact” studies that have examined similar issues in other countries. This would help situate the findings within a broader international context and highlight the contribution of this study to the existing literature.*

Contact theory describes a possible influence of immigration on the attitudes of locals towards migrants. We only briefly mention this literature for the sake of completeness and because we refer to it in our IV estimates. Our focus is on the opposite effect, namely the influence of locals' attitudes towards migrants on migrants' immigration behavior. The central argument from migration theory that we refer to here is the discussion of (dis)amenities as a motive for migration (with xenophobic attitudes as local disamenity).

We regret not having been precise here and hope that our revision of Section 3 will lead to better understanding.

*4. While the use of fixed effects and IV estimation is commendable,* *the paper should provide a more detailed justification for the choice of instruments and discuss their validity in depth. The section on instrumental variables and their explanation in the analysis seem a little bit complex for any reader. These parts could be simplified while retaining the main points.*

Many thanks for this suggestion. We revised the paragraphs on the instruments and extended the discussion of the theoretical arguments behind the instrument variables in section 4 to explain the mechanisms that are supposed to be at work in more detail. Moreover, we moved most of the information on the econometric tests applied to investigate the relevance and exogeneity of the instruments from section 4 and section 5 to the Supporting Information and discuss these results there in more detail. We hope that this reduces the complexity of these paragraphs while at the same time strengthening the arguments for the validity of the instruments.

*5. The analysis of heterogeneous effects across skill groups is a valuable contribution. However, the findings are described as ambiguous. It would be helpful to delve deeper into these results to provide a more nuanced interpretation and possibly identify factors that contribute to the observed differences.*

The inconclusive results are an interim result - and you rightly point to the need for further in-depth research, which might help to understand the factors behind the observed differences. We would like to take this advice on board for our further work. Unfortunately, we are not yet able to implement it in this paper due to data restrictions. However, we discuss the need for further research in the final chapter of the revised paper.

*6. The control variables mentioned in Supporting Info 1 and Supporting Info 2 tables (also referenced in the text) are not shown in the regression models. There are sections in the analysis that refer to these control variables (e.g., labor market competition). Clearly displaying the effects of these control variables both in the tables and in the explanatory sections of the analysis will contribute to the readability of the article.*

Thanks for this suggestion. The Tables A3 and A4 in the Supporting Information S3 and S4 show the results for all control variables of the fixed effects IV estimations. Maybe this was not clearly communicated in the previous version of the paper. We prefer to exclude these results from the main Tables 1 to 3 because these tables already contain a lot of information and might become confusing when 27 more coefficients and standard errors are added for 5 different models. Moreover, we think that discussing all the effects of the control variables and comparing effects across different skill groups will not increase the readability of the paper but distract the reader from the main findings, i.e. how xenophobic behavior affects the immigration rate. However, we now provide a summary of the main results for the control variables together with Table A3 in the Supporting Information S3 and refer to the tables and the corresponding discussion of the control variable results in the first paragraph of Section 5. Please note that labor market competition mentioned in the comment above does not refer to a control variable but one of the instruments.

*7. As stated in the first point, because the hypotheses are not clearly presented, the arguments in the discussion section about whether the hypotheses are supported or not seem somewhat vague.*

We believe that we have resolved this ambiguity by naming the hypotheses, including Table 4, and referring them in the discussion section.

*8. Strengthen the conclusion by summarizing the key findings and their implications more explicitly.* *Highlight the study's contributions and suggest directions for future research.*
The suggestion “Use of hypotheses”, which we followed in section 3 was also helpful to improve the summary of our main results in the last section. We also discuss some issue for future research in the final chapter.

**Reply to Reviewer #2**

We are very grateful for the helpful comments of reviewer 2 for improving our paper. We have taken these remarks into account and have substantially revised our paper. In the following, we describe in detail how we dealt with your comments. We first repeat each comment in italic, black script and then answer in blue script.

- *Lines 16 ff lines 44ff: The authors use the terms “xenophobic attitudes” and “xenophobic behaviour” quite interchangeable. They should say, just in the beginning, if “xenophobic attitudes” relate to “right wing voting” and “xenophobic behaviour” to “xenophobic violence”. Personally, I would prefer the terms “right wing voting” and “xenophobic violence” because these describe what the authors use as indicators.*

So far, we have used the terms xenophobic attitudes and xenophobic behavior interchangeably. After a critical discussion, we believe that “xenophobic attitudes” is the umbrella term that includes “xenophobic behavior”. In literature, both voting behavior and xenophobic violence are used to investigate the development and effects of xenophobic attitudes (e.g. Entorf and Lange 2023, Falk et al. 2011). We decided to use the term xenophobic attitudes strictly to prevent misunderstandings. With this in mind, we also changed the title of the paper. We thank you very much for pointing out our inaccuracy.

We added some sentences to the introduction in which we argue, with reference to the literature, that votes for right-wing parties and xenophobic violence can be used as proxies for right-wing attitudes:

“This might affect regions very differently because stereotypes and prejudices have a strong local dimension, as they contribute to the community culture, which defines the broader societal traits and relations that shape places in terms of the prevailing mindset and overall way of life (Denti et al. 2023, Zajak et al. 2024). […] This paper investigates the effects of regional differences in xenophobic attitudes, measured by votes for right-wing parties in federal elections and xenophobic violence, on the immigration of foreign workers to German district regions. The electoral success of right-wing parties is used as a proxy for xenophobic attitudes, as there is evidence that anti-immigration sentiments are highly correlated with right-wing party support (Arzheimer and Berning 2019) and that the results of national elections reflect local values and attitudes (Bellodi et al. 2024). Our data on xenophobic violence should also indicate the extent of xenophobic attitudes in a region as it is based on regional information from the German security authorities, which records crimes with racist, xenophobic or anti-Semitic motives.”

- *Line 53 or elsewhere: For the international readership the authors should say little bit more about what a German county is. See also line 283*

To avoid misunderstandings, we now consistently write about districts and district regions.

We revised the information on the structure of German local authorities and the district regions to be more precise and added a more detailed description in section 4.1.

- *Lines 74 - 84: I am astonished that the authors present a summary of their results already here. Unusual.*

We have deleted the preview of the results in the introduction.

- *Lines 101: A word about the number of people living in West- and East-Germany would be informative.*

We added the information in section 2.

- *122: Give a description of “political motivated crime” according to the “polizeiliche Kriminalstatistik”. Here or in the discussion section the authors should also discuss the low reliability and validity of the indicator (which might also help to understand some of the results related to this indicator). See also 308.*

Many thanks for this suggestion. The definition of political motivated crime (from the right) is given at the beginning of the section “Main explanatory variables: indicators for xenophobic attitudes”. In the revised version of the paper, we provide a more detailed description of the statistic here. Moreover, we added a brief discussion of related measurement issues and the validity of the data. However, we do not agree that the reliability of the indicator is basically low, but explain that measurement problems might affect the results in the discussion of the regression results in section 5.

- *151: A summary of the up-to-date state of contact theory is* *Pettigrew, T. F., & Tropp, L. R. (2011). When groups meet: The dynamics of intergroup contact. Psychology Press.*

Thank you. We added the reference.

- *316: Mentioning the percentages of votes for the different parties received in the last election would give an impression of the dominance of the AfD in this context.*

We added information about the dominance of the AfD in section 2.

- *324 ff: “In some studies, the variables are negatively correlated, which is attributed to an opportunity effect caused by the existence of right-wing parties [62].” A short explanation would be helpful.*

We changed the sentence as follows:

In some studies, the variables are negatively correlated, which is attributed to an opportunity effect. Accordingly, people are less likely to commit right-wing extremist attacks where right-wing parties represent a relevant force within the political system and thus an alternative way of exerting pressure on political elites (Koopmans 1996).

- *330: The naming of the size of the correlation for the study at hand here is unusual. I recommend moving it to the results section.*

We moved the sentence to section 5.

- *336 f: Adding the size of the different groups would be helpful.*

We added S 8 Table A8 with summary statistics on the size of the different immigrant groups in the Supporting Information and refer to this table in the paragraph on coethnic communities in section 4.1.

- *369 ff: Why did the authors use the LOGARITHMS of ratios?*

Using logarithms of the dependent and the explanatory variables in the model is a rather common approach in migration research (see e.g. Gorinas and Pytliková 2017, Heider et al. 2020 or Mitze, 2019). This specification has also the advantage that all coefficients can be easily interpreted as elasticities, i.e. they give the percentage change of the immigration rate for a change of the corresponding explanatory variable by 1%. We added this information in section 4.2 below equation (2). However, we refrain from a more detailed discussion of econometric advantages of this specification (e.g. measurement errors and outliers become less important).

- *478 ff: The authors should remind the reader why they could not analyse effects of right-wing votes and xenophobic violence simultaneously.*

Many thanks for this suggestion. We added an analysis where right-wing votes and xenophobic violence are both included in the model. We briefly discuss the results in section 5. Table A7 with the results is included in the Supporting Information S_7. The results are qualitatively in line with our main results, i.e. the coefficients of the variables are negative with the absolute size being larger and increasing for the right-wing votes in the extended model. While the adverse effect of the right-wing votes is thus confirmed by the extended model, evidence on an important effect of xenophobic violence is less robust. However, including both variables in one model comes at some costs. The sample size strongly declines because there is no perfect overlap of the two variables. The number of observations for the model including the right-wing votes only is 1,050. If we add the variable for xenophobic violence, the number of observations declines to 488. The reduction of the sample size affects the precision of estimates and as a result standard errors increase significantly.

- *609: The authors refer to columns in tables. Printing the column number in the head of the tables would be helpful.*

We added the column numbers in the tables.

**Literature**

Arzheimer K, Berning C. How the Alternative for Germany (AfD) and their voters veered to the radical right, 2013–2017. Electoral Studies. 2019;60(102040). DOI: 10.1016/j.electstud.2019.04.004.

Bécares L, Nazroo J, Stafford M. The buffering effects of ethnic density on experienced racism and health. Health & Place. 2009;15:700-708. DOI: 10.1016/j.healthplace.2008.10.008.

Bendix J, Liebler CM. Place, Distance, and Environmental News: Geographic Variation in Newspaper Coverage of the Spotted Owl Conflict. Annals of the Association of American Geographers.1999;89(4): 658-676. https://doi.org/10.1111/0004-5608.00166.

Biskamp F. A Societal Shift to the Right orthe Political Mobilisation of a Shrinking Minority? Explaining Rise and Radicalisation of the AfD in Germany. International Journal of Public Policy. 2024;17(3):139–165. DOI: 10.1504/IJPP.2024.138373.

Bellodi, L., Docquier, F., Iandolo, S., Morelli, M. & Turati, R. (2024). Populism, Selective Mobility, and the Political Polarization of Italian Municipalities. IZA Discussion Paper No. 16732.

Branton RP, Dunaway J. Spatial Proximity to the U.S.—Mexico Border and Newspaper Coverage of Immigration Issues. Political Research Quarterly. 2009; 62(2):289-302. DOI: 10.1177/1065912908319252.

Denti D, Crociata A, Faggian A. Knocking on Hell’s door: dismantling hate with cultural consumption. Journal of Cultural Economics. 2023;47(2): 303–349. DOI: 10.1007/s10824-022-09461-8.

Entorf H, Lange M. Refugees welcome? Understanding the regional heterogeneity of anti-refugee hate crime. Regional science and urban economics. 2023;101,103913. DOI: 10.1016/j.regsciurbeco.2023.103913.

Falk A, Kuhn A, Zweimüller J. Unemployment and Right-wing Extremist Crime. Scandinavian Journal of Economics. 2011; 113: 260-285. DOI: 10.1111/j.1467-9442.2011.01648.x.

Fennema M. The concept and measurement of ethnic community. Journal of Ethnic and Migration Studies. 2004;30(3), 429-447. DOI: 10.1080/13691830410001682025.

Gorinas C, Pytliková M. The Influence of Attitudes Toward Immigrants on International Migration. International Migration Review. 2017;51(2):416-51. DOI: 10.1111/imre.12232.

Heider B, Stroms P, Koch J, Siedentop S. Where do immigrants move in Germany? The role of international migration in regional disparities in population development. Population, Space and Place. 2020;26(8):1-19. DOI: 10.1002/psp.2363.

Ireland PR. Socialism, Unification Policy and the Rise of Racism in Eastern Germany. International Migration Review. 1997;31(3): 541-68. DOI: 10.2307/2547285.

Knabe A, Rätzel S, Thomsen SL. Right-Wing Extremism and the Well-Being of Immigrants. Kyklos. 2013;66(4):567-90. DOI: 10.1111/kykl.12037.

Koopmans R. Explaining the Rise of Racist and Extreme Right Violence in Western Europe: Grievances or Opportunities? European Journal of Political Research. 1996;30:185-216. DOI: 10.1111/j.1475-6765.1996.tb00674.x.

Mitze T. The Migration Response to Local Labour Market Shocks: Evidence from EU Regions During the Global Economic Crisis. Oxford Bulletin of Economics and Statistics. 2019;81(2):271-98. DOI: 10.1111/obes.12271.

Pettigrew TF, Tropp LR. When groups meet: The dynamics of intergroup contact. New York: Psychology Press; 2011. DOI: 10.4324/9780203826461.

Shankley W, Laurence J. Community ethnic density, ethnic segregation, and ethnic minorities' common mental disorders in the UK. Health & Place. 2022;73. DOI: 10.1016/j.healthplace.2021.102723.

Weisskircher M. The strength of far‐right AfD in eastern Germany: The east‐west divide and the multiple causes behind ‘populism’. The Political Quarterly. 2020;91(3):614-622. DOI: 10.1111/1467-923X.12859.

Zajak S, Best F, Pickel G, Quent M, Römer F, Steinhilper E, Zick A. Ablehnung, Angst und Abwanderungspläne: Die gesellschaftlichen Folgen des Aufstiegs der AfD. [Internet]. Berlin: Deutsches Zentrum für Integrations- und Migrationsforschung (DeZIM); 2024 [cited 2024 Oct 24]. Available from: https://www.dezim-institut.de/publikationen/publikation-detail/ablehnung-angst-und-abwanderungsplaene/.
